# Identification of novel probiotic lactic acid bacteria from soymilk waste using the 16s rRNA gene for potential use in poultry

**DOI:** 10.14202/vetworld.2024.1001-1011

**Published:** 2024-05-09

**Authors:** Anifah Srifani, Mirnawati Mirnawati, Yetti Marlida, Yose Rizal, Nurmiati Nurmiati, Kyung-Woo Lee

**Affiliations:** 1 PMDSU Program, Graduate Program of Animal Feed and Nutrition Department, Faculty of Animal Science, Universitas Andalas, Padang, West Sumatera, Indonesia; 2 Department of Animal Feed and Nutrition, Faculty of Animal Science, Universitas Andalas, Padang, West Sumatera, Indonesia; 3 Department of Biology, Faculty of Mathematics and Natural Sciences, Universitas Andalas, Padang, West Sumatera, Indonesia; 4 Department of Animal Science and Technology, Sanghuh College of Life Sciences, Konkuk University, Seoul, South Korea

**Keywords:** lactic acid bacteria, poultry, probiotic

## Abstract

**Background and Aim:**

In-feed antibiotics have been used as antibiotic growth promoters (AGPs) to enhance the genetic potential of poultry. However, the long-term use of AGPs is known to lead to bacterial resistance and antibiotic residues in poultry meat and eggs. To address these concerns, alternatives to AGPs are needed, one of which is probiotics, which can promote the health of livestock without having any negative effects. *In vitro* probiotic screening was performed to determine the ability of lactic acid bacteria (LAB) isolated from soymilk waste to be used as a probiotic for livestock.

**Materials and Methods:**

Four LAB isolates (designated F4, F6, F9, and F11) isolated from soymilk waste were used in this study. *In vitro* testing was performed on LAB isolates to determine their resistance to temperatures of 42°C, acidic pH, bile salts, hydrophobicity to the intestine, and ability to inhibit pathogenic bacteria. A promising isolate was identified using the 16S rRNA gene.

**Result:**

All LAB isolates used in this study have the potential to be used as probiotics. On the basis of the results of *in vitro* testing, all isolates showed resistance to temperatures of 42°C and low pH (2.5) for 3 h (79.87%–94.44%) and 6 h (76.29%–83.39%), respectively. The survival rate at a bile salt concentration of 0.3% ranged from 73.24% to 90.39%, whereas the survival rate at a bile salt concentration of 0.5% ranged from 56.28% to 81.96%. All isolates showed the ability to attach and colonize the digestive tract with a hydrophobicity of 87.58%–91.88%. Inhibitory zones of LAB against pathogens ranged from 4.80–15.15 mm against *Staphylococcus aureus*, 8.85–14.50 mm against *Salmonella enteritidis*, and 6.75–22.25 mm against *Escherichia coli*. Although all isolates showed good ability as probiotics, isolate F4 showed the best probiotic ability. This isolate was identified as Lactobacillus casei strain T22 (JQ412731.1) using the 16S rRNA gene.

**Conclusion:**

All isolates in this study have the potential to be used as probiotics. However, isolate F4 has the best probiotic properties and is considered to be the most promising novel probiotic for poultry.

## Introduction

The poultry industry is one of the most crucial industries in Indonesia because it can produce animal proteins for human consumption and increase employment opportunities. One of the problems faced by the poultry industry is disease interference caused by pathogens. *Salmonella enteritidis*, *Escherichia coli*, and *Staphylococcus aureus* are pathogens that can cause disease in broiler chickens [1]. The use of in-feed antibiotic growth promoters (AGPs) has been widely used to overcome diseases and stimulate growth and productivity of poultry. The use of AGPs in poultry has led to the occurrence of resistant bacteria and, consequently, negative effects on humans [2]. In addition, dietary antibiotics can disrupt the balance of micro-organisms in the digestive tract of humans and animals [3]. The long-term use of antibiotics may jeopardize human health, including the resistance of pathogenic bacteria to antibiotics and the accumulation of antibiotic residues in animal meat. In Europe, the use of AGPs as feed additives was gradually limited from 1970 to 2003 and was banned on January 01, 2006 [4]. In Indonesia, Animal Husbandry and Animal Health Law No. 18 of 2009, Article 22 Paragraph 4c, bans the use of antibiotics as feed additives [5]. Therefore, potential alternatives to in-feed AGPs, including probiotics, prebiotics, enzymes, and organic acids, have been proposed [6].

Probiotics are defined as live micro-organisms that have a beneficial effect on the health of the host upon ingestion. The criteria for probiotic micro-organisms that have been established by the Food and Agriculture Organization/World Health Organization [7] include that they have to be able to survive in conditions of stomach acid and digestive bile salts, provide benefits to the intestines, be able to stick to mucus and/or intestinal epithelial cells, produce antimicrobial activity against pathogenic bacteria, are micro-organisms which are safe or include generally recognized as safe micro-organisms, do not excogitate toxins, are not resistant to antibiotics, and are not pathogenic bacteria. Probiotics are mostly isolated from fermented dairy products or the human gastrointestinal tract [8]. In addition, many studies have investigated potential micro-organisms from non-traditional sources such as traditional fermented foods, beverages, vegetables, vegetable wastes [9–11], and local foods [12]. Lactic acid bacteria (LAB) are considered the most studied probiotic bacteria. LAB is well-known for its ability to withstand the intestinal environment and produce digestive enzymes (i.e., amylase, chitinase, lipase, phytase, and protease) that can enhance digestion in the gastrointestinal tract [13]. LAB plays a number of roles, particularly in encouraging the immune system and enhancing growth performance [14–16]. LAB can balance innate immunity and modulate gut homeostasis [17]. LAB can also produce various metabolites, such as lactic acid, antioxidants, and antimicrobial compounds, through carbohydrate fermentation in the intestine, particularly bacteriocins and short-chain fatty acids (SCFA), which can deter pathogenic bacteria growth [18].

Soy milk waste has the potential to be used as poultry feed because of its high protein content. However, the use of soy milk waste is still hindered by its high cellulose and phytic acid content. Cellulose is naturally degraded by cellulase, whereas phytic acid can be degraded by phytase. Previous research by Srifani *et al*. [19] has succeeded in isolating 13 LAB that can produce cellulase and phytase enzymes from soy milk waste. Of these 13 isolates, four were selected based on their highest enzyme activity. Four isolates were designated as isolates F4, F6, F9 and F11. These isolates could produce cellulase enzymes of 17.69, 20.67, 14.72, and 13.13 U/mL, respectively [19]. Successful isolation of LAB producing cellulase and phytase enzymes from soy milk waste showed that these bacterial isolates may produce cellulase to hydrolyze cellulosic materials and phytases to hydrolyze phytic acid. Therefore, this study aimed to discover novel poultry probiotics from LAB isolates that had previously been isolated from soy milk waste. Thus, this research reveals novel LAB strains with high probiotic properties that can potentially be used in poultry and commercialized for poultry farmers in and outside Indonesia.

## Materials and Methods

### Ethical approval

This study does not entail ethical approval because it did not use humans or animals as research subjects.

### Study period and location

This study was conducted from May to July 2023 in the Feed Industry Technology Laboratory, Non-Ruminant Nutrition Laboratory, Animal Biotechnology Laboratory, Andalas University, and the Bacteriology Laboratory of Bukittinggi Veterinary Center.

### LAB

This study used four LAB isolates previously isolated from soy milk waste [19].

### Resistance of LAB to 42°C

A modified method from Kumar *et al*. [9] was used to test LAB resistance at 42°C. All test bacterial isolates were grown on De Man, Rogosa, and Sharpe Agar (Merck KGaA, Darmstadt, Germany) media and incubated at 42°C for 24–48 h.

### Gastric pH survivability

The survivability test for gastric pH was performed based on the modified method from Dowarah *et al*. [20]. De Man, Rogosa, and Sharpe Broth (MRSB) (Merck KGaA) was added with 37% HCl (Merck KGaA) to obtain a pH of 2.5. MRSB without HCl was used as a control (a pH of 6.8). The mediums were autoclaved at 121°C for 15 min. Approximately 0.5 mL of LAB isolates was added to 5 mL of MRSB-HCl and incubated for 3 and 6 h at 37°C. The absorbance was read at 600 nm. The analysis was conducted with three repetitions. Gastric pH survivability can be calculated as follows [21]:


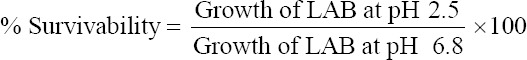


### LAB tolerance to bile salt

We tested bile salt tolerance using a modified method from Nwachukwu *et al*. [22]: The concentrations of salt used in MRSB were 0%, 0.3%, and 0.5%. Subsequently, the media were autoclaved for 15 min at 121°C. After adding approximately 0.5 mL (10^10^ colony forming unit [CFU]/mL) of isolates to 5 mL of prepared MRSB (Merck KGaA) plus 0%, 0.3%, and 0.5% of oxgall (Sigma-Aldrich, St. Louis, MO, USA), the mixture was incubated for 5 h at 37°C. MRSB (Merck KGaA) with no oxgall addition (0% concentration) was used as a control. Lab growth measurements were made using absorbance measurements at 600 nm. Three repetitions of this were made. According to Tokatli *et al*. [21], it can be calculated as follows:


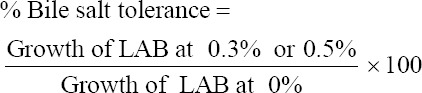


### Hydrophobicity of LAB

The stainless steel was cleaned and 0.68 g of MRSB (Merck KGaA) was dissolved in 100 mL of distilled water. The media were then autoclaved at 121°C for 15 min. A total of 1 mL of LAB isolate was added to the media, followed by stainless-steel incubation for 24 h at 37°C. A swab was applied to the stainless-steel surface. This swab was placed in a tube containing 10 mL of phosphate buffer (Merck KGaA), homogenized, and measured at a wavelength of 600 nm (A). Liquid phase growth measurements were performed by taking 1 mL of liquid from the MRSB (Merck KGaA) medium and diluting it into 9 mL of phosphate-buffered saline (Merck KGaA). We measured the absorption at a wavelength of 600 nm (Ao). According to Fadda *et al*. [23], hydrophobicity was calculated using the following formula:


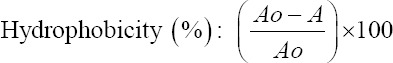


Ao: OD value in the liquid phase, A: OD value attached to *stainless steel*

### Inhibition test to pathogen

Inhibition tests against pathogens were performed according to the modified method of Baqis *et al*. [24]. NA (Merck KGaA) medium was prepared and autoclaved for 15 min. The cooled media were added to 0.2% pathogenic bacteria, homogenized, and poured into a Petri dish. Paper disks were soaked in MRSB (Merck KGaA) containing the isolate for 10 min. A paper disk was then placed on the surface of the NA medium (Merck KGaA) and incubated for 24 h at 37°C. Calipers were used to measure the diameter of the inhibition zone. Calculation of the inhibition zone was performed based on Winastri *et al*. [25].

### Molecular identification

On the basis of the probiotic test, we selected one of the best isolates with high probiotic characteristic ability and performed a molecular identification test on it. It was performed using the following steps.

### Genomic DNA Isolation

The previously harvested bacterial pellets were added to Lysis Solution (Merck KGaA), Proteinase K (Merck KGaA), and homogenized. The microtubes were incubated at 56°C for 30 min. RNAse A solution (Merck KGaA) was vortexed and incubated at room temperature for 10 min, followed by the addition of 50% cold ethanol. The solution was transferred to a PC and centrifuged at 8,000× *g* for 1 min. The PC was moved to the collection tube, where Wash Buffer I was added. After discarding the solution inside the collection tube, PC was added, the collection tube was centrifuged, and Wash Buffer II was added. Elution buffer (Merck KGaA) (approximately 50 μL) was added in the center of the PC. It was then incubated for 2 min at 28°C and centrifuged for 1 min at a speed of 13,000× *g*. The microtube containing the DNA solution was then stored at –20°C after the PC was disposed.

### Electrophoresis of DNA

Approximately 1% of agarose gel was prepared by adding 0.5 g of agarose powder (Promega, Madison, USA) to a Schott bottle. Subsequently, 50 mL of Tris Borate EDTA (TBE) buffer was added. The bottle was heated to medium heat for 3 min. Ethidium bromide (Merck KGaA) (approximately 5 μL) was introduced and thoroughly mixed. Gel trays and combs were affixed to the agarose molds, taking into account the number of samples processed. The agar solution was then dispensed into the gel tray and allowed to solidify. A total volume of 10 μL was used to create the electrophoretic cocktail composition, which included 2 μL of genomic DNA, 1 μL of 10× bromo phenol blue, and 7 μL of 1× Tris-EDTA. Similarly, the cocktail composition for the λ DNA marker (50 ng/μL) was prepared. As soon as the agarose gel solidified, the comb was removed and the gel tray was detached from the mold. The electrophoresis device is set up by connecting the chamber to the power supply. The gel tray was positioned inside the electrophoresis chamber, and TBE buffer was poured in until the gel surface was fully submerged. DNA samples were meticulously loaded into individual wells of a gel, commencing with the λ DNA marker in the leftmost well, followed by genomic DNA samples. Electrophoresis was performed at a voltage of 100 V for 30 min. A Gel Documentation System (Biometra, Germany) equipped with an ultraviolet (UV) transilluminator was used to visualize and document the gels.

### Genomic DNA quantification (Biodrop, Cambridge, UK)

As much as 1 μL of DNA sample was taken, the tip was placed directly between the detector wire on the biodrop (Biodrop), and measurements were taken on the Biodrop UV-visible spectrophotometer.

### DNA amplification (polymerase chain reaction [PCR])

A sterile 0.2 mL microtube was used to prepare the PCR mixture. We added 25 μL of KOD One Blue Mastermix (Toyobo, Japan), 2 μL of Primer 16SrRNA_27F (10 ng/μL), 2 μL of Primer 16SrRNA_1525R (10 ng/μL), 2 μL of DNA bacterial genome (10 ng/μL), and 19 μL of nuclease-free water to the microtube. The microtubes were then placed in a PCR machine that matched the characteristics of the 16SrRNA primer. After PCR amplification, samples were separated by electrophoresis on a 1% agarose gel. Two microliters of a 1 kb marker was loaded into the leftmost well, followed by 5 μL aliquots of each PCR reaction mixture loaded into adjacent wells. Microtubes containing the remaining PCR reaction mixtures were stored at –20°C for future use. The agarose gels were visualized and documented using a gel documentation system.

### Statistical analysis

All experiments were performed in triplicate, and the results are expressed as the mean ± standard deviation [26]. The data obtained were statistically analyzed using IBM Statistical Package for the Social Sciences Statistics 26.0 version (IBM Corp., NY, USA). One-way analysis of variance was used to study significant differences between means, with a significance level of p = 0.05. Duncan’s multiple ranges test was used to discriminate differences between means. Differences were considered statistically significant at p < 0.05.

## Results

### Resistance of LAB at 42°C

**Figure-1:**
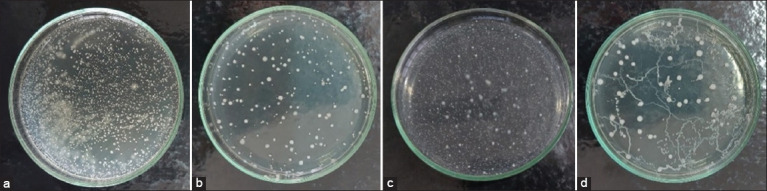
Resistance of lactic acid bacteria isolates at an incubation temperature of 42°C, (a) isolate F4, (b) isolate F6, (c) isolate F9, (d) isolate F11.

Figure-1 shows the effect of an incubation temperature of 42°C on bacterial growth. On the basis of the research results, all tested LAB isolates showed resistance to 42°C. The growth of LAB isolates on MRS agar media after incubation can be observed. In this study, isolate F4 showed better growth than the other three LAB isolates.

### Resistance (%) of LAB to gastric pH

Resistance to gastric juice with a pH of 2.5 after 3 and 6 h of incubation is shown in Table-1. All LAB isolates were resistant to a pH of 2.5 for both 3 and 6 h. Isolate F4 had the highest resistance and significantly differed from isolates F6, F9 and F11 after 3 and 6 h of incubation.

**Table 1:** Resistance of LAB to gastric pH.

Isolate	Time	

	3 h	6 h
F4	94.44 ± 2.686^c^	90.86 ± 0.749^d^
F6	79.87 ± 1.391^a^	76.29 ± 0.895^a^
F9	90.33 ± 1.202^b^	82.90 ± 1.627^c^
F11	91.58 ± 0.816^b^	80.15 ± 1.325^b^

*Values are means and standard errors of three replicates. Means in the same column with different letter are significantly different (p < 0.05). LAB=Lactic acid bacteria

### Resistance of LAB to bile salt (%)

Table-2 shows the resistance of LAB isolates to 0.3% and 0.5% bile salts. All LAB isolates showed resistance of more than 70% at an oxgall salt concentration of 0.3% and resistance of more than 50% at an oxgall salt concentration of 0.5%. Isolate F11 was found to have the highest resistance to 0.3% and 0.5% bile salts, whereas isolate F11 did not differ significantly from isolate F4 but was significantly different from isolates F6 and F9.

**Table 2:** Resistance of LAB to bile salt.

Isolate	Resistance (%)	

	0.3	0.5
F4	88.07 ± 1.967^c^	81.24 ± 2.281^c^
F6	82.85 ± 2.222^b^	72.84 ± 2.687^b^
F9	73.24 ± 1.719^a^	56.28 ± 2.460^a^
F11	90.39 ± 4.082^c^	81.96 ± 0.704^c^

*Values are means and standard errors of three replicates. Means in the same column with different letter are significantly different (p < 0.05). LAB=Lactic acid bacteria

### The hydrophobicity of LAB

Table-3 shows the hydrophobicity of LAB isolates in this study. The results show that isolate F6 has the highest hydrophobicity and isolate F9 has the lowest hydrophobicity.

**Table 3:** Hydrophobicity of LAB.

Isolate	LAB hydrophobicity (%)
F4	91.24 ± 0.906
F6	91.88 ± 1.799
F9	87.58 ± 4.677
F11	89.34 ± 3.060

*Values are means and standard errors of three replicates. Values are not significantly different (p > 0.05). LAB=Lactic acid bacteria

### Inhibition of pathogenic bacteria by LAB

**Figure-2:**
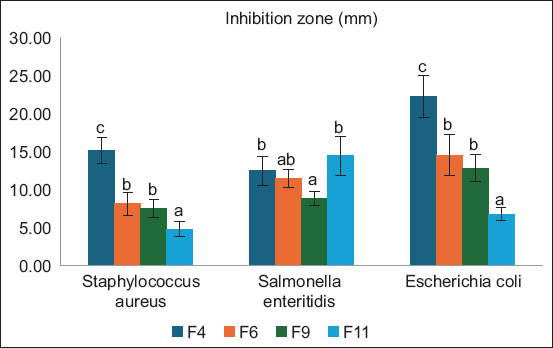
Inhibitory diameter against pathogenic bacteria. *Values are means and standard errors of three replicates. Different letters show significant differences (p < 0.05).

Figure-2 shows the laboratory inhibition zone against pathogens. Each isolate has a different inhibitory zone against each type of pathogen.

### Molecular identification of selected LAB isolates

**Figure-3:**
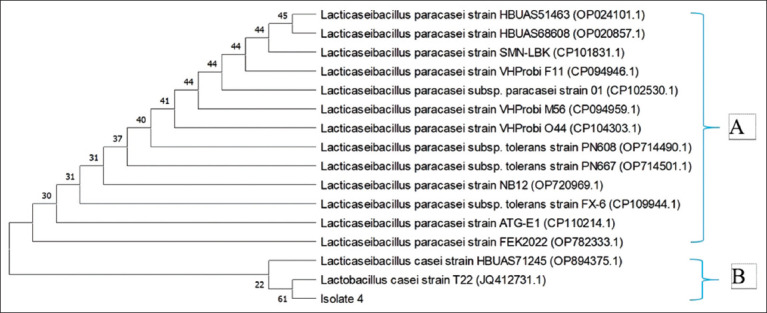
The phylogenetic tree of lactic acid bacteria based on 16S rRNA gene sequences.

The Neighbor-Joining method was used to create the phylogenetic tree using a bootstrap value of 1000. To examine evolutionary distances, the Kimura two-parameter approach was employed. Based on the 16S rRNA gene sequence, Figure-3 displays a phylogenetic tree that illustrates the relationship between isolate F4 bacterium and the 15 reference bacteria from the GenBank.

## Discussion

### Resistance at the temperature of 42^o^C

The resistance of LAB isolates to heat was determined at 42°C, which is the average temperature of the chicken body and digestive tract [27, 28]. It is recommended that probiotic bacterial isolates should be able to live and survive at this temperature. Bacteria in the environment are exposed to a series of variable factors, one of which is a large temperature change that allows them to live and withstand certain temperature ranges. Microbes can be classified as psychrotrophs, mesophiles, and thermophiles on the basis of their ability to survive at different temperatures, whose tolerances include the following temperature ranges: 2°C–7°C, 10°C–40°C, and 43°C–66°C, respectively [29]. Another opinion stated that mesophiles and thermophiles can live at temperatures of 23°C–45°C and 45°C–65°C, respectively [30]. LAB isolates in this study can be categorized as mesophile bacteria because they can live at a temperature of 42°C (Figure-1). Thus, all the LAB isolates tested can live at body and digestive temperatures in chickens, so they have the potential to be tested further as probiotics.

### LAB resistance to gastric pH

Resistance testing of LAB isolates was performed at pH 2.5. This is because the pH of proventriculus and gizzard in chickens is 2.5 [31]. The pH in gizzards ranges between 2.5 and 3.5 [32] proventriculus contains hydrochloric acid and pepsinogen. The pH of gastric fluid secreted from the proventriculus is reported to be approximately 2 [33]. A low pH of 2 automatically activates pepsinogen to produce pepsin, a protease with an optimal acidic pH [34]. Pepsin plays an important role in the digestion of first-line feed during feed retention in poultry [35]. The longest food retention time is estimated to be in the proventriculus and gizzard, between 30 min and 2 h before the partially digested chyme is released into the small intestine [36]. Therefore, during this period, the probiotic isolate should be able to withstand the low pH of the proventriculus and gizzard.

The resistance obtained in this study is more than 70% at low pH (Table-1). These results show that all LAB isolates can be used as probiotics in terms of resistance to acidic pH. Mulaw *et al*. [37] reported that the resistance of LAB isolates to pH 2.5 for 3 h was >50%. Based on the research results in Table-1, isolate F4 was found to have the best resistance compared to the other isolates (94.44% after 3 h of incubation and 90.86% after 6 h of incubation). This resistance by the F4 isolate indicates that LAB isolates are highly resistant to acidic pH. This is in accordance with Skenderidis *et al*. [38], who reported that good probiotics have high survival and are less affected by acidic pH.

The resistance to low pH obtained in this study was higher than that reported by Ribeiro *et al*. [39], who reported a survival rate of 45.9% at pH 2.5 after 6 h of incubation for the test isolate (*Pediococcus acidilactici* B14). Lie *et al*. [40] reported that the *Lacticaseibacillus rhamnosus* Probio-M9 strain had a survival rate of 83.72% at pH 2.5 after 3 h of incubation. Vijayalakshmi *et al*. [41] reported survival rates of *Lactobacillus helveticus* IDCC3801, *Lactiplantibacillus plantarum* BNH17, and *L. rhamnosus* IDCC3201 of 92.83%, 90.50%, and 59.56% at pH 2 after 3 h of incubation, respectively. At a pH of 2.5, Ahmed *et al*. [42] reported a survival rate of 81.68%–85.01% after 3 h of incubation for the 16 bacterial isolates.

Acid tolerance tests are carried out to imitate the gastric conditions of poultry more closely. To reach the intestine while alive, micro-organisms pass through the gastrointestinal tract of poultry, since they are ingested in the mouth until they reach the intestine. Tolerance to acids and bile is considered a prerequisite property for potential probiotics. Gastric juice can reduce microorganism colonies due to its low pH (approximately 2). Therefore, it is essential that they have protection systems to withstand the wide range of pH experienced during digestion. LAB is a microorganism that has the ability to tolerate acidic pH [43]. LAB has three main defense systems to survive low pH conditions: H+-ATPase proton pump, arginine deaminase, and glutamate decarboxylase systems [44]. Furthermore, Guo *et al*. [45] claimed that micro-organisms can defend themselves from low pH through direct active proton extrusion, decarboxylase-mediated proton consumption, alkaline substances production, cell membrane modification, repair and damage avoidance of macromolecules, and reconstruction of metabolic pathways.

All LAB isolates used in this study were previously characterized by Srifani *et al*. [19] and found as gram-positive bacteria. Gram-positive bacteria have a peptidoglycan thickness of tens of nanometers, which is generally described as a homogeneous structure that provides mechanical strength [46]. Gram-positive bacteria have thick cell walls composed mainly of proteins, peptidoglycans, and teichoic acids (TA). TA is responsible for the initial surface attachment of cells. When TA is attached to the cell wall, it is called wall TA, and when it is bound to the cell membrane, it is called lipo TA [47]. TA contributes up to 50% of cell wall mass and helps maintain cell cation homeostasis. According to Harnentis *et al*. [1], peptidoglycan and TA can preserve cell wall shape even in low pH conditions.

### LAB resistance to bile salt

Resistance to bile salts is an important probiotic candidate criterion because bile salts are strong surfactants, and exposure to bile in the digestive tract is very toxic for bacterial species to survive and maintain activity in the intestine [48]. Probiotics must withstand complex conditions in the gastrointestinal tract, including the presence of bile. Bile has antimicrobial properties and plays a significant role in the physicochemical defense system of the body [49]. Bile may cause damage to the bacterial membrane. Probiotics must be resistant to bile salts so that they can survive in the intestine and play a functional role as probiotic. High bile salt resistance in a bacterial isolate makes it easier to colonize the host’s digestive tract [50]. Therefore, it is important to assess the potential ability of probiotics to grow in the presence of bile salts.

LAB, which has good resistance to bile salts, has a high potential to be used as a probiotic because it can survive in the digestive tract. The bile salt concentration in the digestive tract ranges from approximately 0.2% to 0.3% depending on the host and the type and amount of feed consumed [51]. A bile salt concentration of 0.3% is considered a critical limit for the selection of probiotics. In this study, we tested the resistance of LAB isolates to 0.3% and 0.5% oxgall salt concentrations (Table-2).

According to Liu *et al*. [52], bacteria are categorized as having resistance to bile salts if they have a survival rate above 50% at a certain bile salt concentration. Kaewarsar *et al*. [53] also reported that LAB isolates as a probiotic had >50% resistance to low pH gastric juice and 0.3% bile salt concentration after 4 h of incubation. In this study, all LAB isolates were classified as good probiotic candidates because they exhibited bile salt resistance of more than 50% at bile salt concentrations of 0.3% and 0.5%.

Isolate F11 showed the highest resistance, 90.39% at 0.3% concentration. When the bile salt concentration was increased to 0.5%, the resistance was reduced to 81.96% (a percentage reduction of 8.43%) (Table-2). These results are still higher than those reported by Xu *et al*. [54] reported that 8 LAB isolates isolated from the digestion of local Chinese breed chickens had resistance to bile salts ranging from 30%–80% at a bile salt concentration of 0.3% and 20%–47% at a bile salt concentration of 0.5% after 2 h of incubation. The highest survival rate from this study came from the *Lactobacillus johnsonii* isolate, which had a survival rate of 88.29% at 0.3% bile salts and decreased drastically to 31.91% when the bile concentration increased to 0.5%. One of the rules for testing microbial resistance to bile salts is that increasing the concentration of bile salts will reduce the survival rate of bacteria [55, 56]. High concentrations of bile salts often lead to an increase in the number of dead bacterial cells. Another study reported by Wang *et al*. [57] reported survival rates of 58.38%, 47.13%, and 45.45%, respectively, for three test isolates LP1, WT1, and PT2 isolated from the digestive tract of Tibetan chickens at a bile salt concentration of 0.3% with an incubation period of 18 h. Khurajog *et al*. [58] isolated *L. salivarius* BF12, *P. acidilactici* BF9, *P. acidilactici* BF14, *P. acidilactici* BYF20, and *P. acidilactici* BYF26 from chicken feces and found survival rates of 71.75%, 84.77%, 72.20%, 78.16%, and 82.11% at 0.3% bile salts after 6 h of incubation. These varying results are due to differences in the micro-organisms used. Each LAB isolate exhibits different resistance to bile salts in the digestive tract.

Resistance to bile salts is an important characteristic of probiotic candidates. Bile salts are able to prevent the growth of micro-organisms in various ways. Bile salts can damage the phospholipid structure of organelles and bacterial cell membranes as a fat emulsifier. The results of this study show that all LAB isolates can survive in poultry digestive tract conditions. Resistance to bile salts is mainly due to the presence of bile salt hydrolase (BSH) [59]. This enzyme is a defense mechanism against intracellular acidity caused by conjugated bile salts. BSH hydrolyzes and deconjugates glycine or taurine from the cholesterol cores of bile acids [60]. According to Bustos *et al*. [61], BSH converts deconjugated bile salts into free bile salts (FBS). FBS acts as signaling molecules in various metabolic processes, including the regulation of dietary lipid absorption, cholesterol metabolism, energy homeostasis, and inflammation [62]. Harnentis *et al*. [1] stated that FBSs can play a role in creating homeostatic conditions in bacterial membranes and regulate the ups and downs of nitrogen bases, fats, and amino acid biosynthesis, which influence fat alteration resulting in the production of exopolysaccharides (EPSs). According to Hidalgo-Cantabrana *et al*. [63], EPS can be used by bacteria to protect themselves against the harsh conditions of the gastrointestinal tract, thereby increasing their persistence in the host body. Nambiar *et al*. [64] added that EPS allows bacteria to be more resistant to low pH and bile salts. EPS protects against bile salt concentrations of 0.15%–0.3% at pH 2–3 [65].

### Hydrophobicity of LAB

Table-3 shows that all LAB isolates have hydrophobicity of >85%. The hydrophobicity obtained in this study was higher than that found by Vijayalakshmi *et al*. [41], who found that of the nine isolates tested, the hydrophobicity ranged from 39.18 to 64.98%, with the highest percentage obtained by *L. plantarum* BNH17. Mohammad *et al*. [66] reported hydrophobicities of 59.41%, 52.09%, 74.51%, and 80.52% for isolates *Lactobacillus musae* SGMT17, *Lactobacillus crustorum* SGMT20, *Lactobacillus mindensis* SGMT22, and *Leuconostoc mesenteroides* U39, respectively. Wang *et al*. [67] reported the hydrophobicity of bacterial isolates LSG1-1 and LSG2-1 isolated from the digestive tract of *Rhynchocypris lagowskii*, which had hydrophobicities of 73.44% and 77.81%, respectively.

An important property of probiotic bacteria is their resistance to low pH and bile salts. Another criterion is that the bacterial isolate must be able to attach and colonize the digestive tract. According to Falah *et al*. [68], measurements of hydrophobicity can be considered as an initial test of probiotic ability to bind epithelial cells. Hydrophobicity is considered to be an important feature that enhances the first contact between host cells and probiotic strains. These characteristics make it the most appropriate choice not only to eliminate/reduce pathogen adherence [69], but also to provide health benefits to their respective hosts. Cell surface hydrophobicity is a measure of intestinal colonization, in the form of adhesion and persistence after entering the intestinal cavity [70]. A high percentage of hydrophobicity indicates a higher potential for bacterial attachment in the intestine, resulting in higher colonization [71, 72]. Bacterial isolates with high hydrophobicity have a very strong interaction with mucosal cells. Each bacterial isolate has different hydrophobicity capabilities, which are influenced by the composition and structure of the walls and membranes of each cell. Various nonpolar molecules, such as TA, lipoteichoic acid, lipopolysaccharide, and several surface proteins (S layer) on the cell surface play an important role in hydrophobic behavior [73]. In addition, EPS influence the adhesion of bacteria to host cells [74]. No fixed molecule can be applied to all probiotic strains, although there are many adhesion-related molecules. Many adhesins appear to be species- or strain-dependent.

### LAB inhibition to pathogenic bacteria

Figure-2 shows LAB inhibition to pathogenic bacteria (*S. aureus, S. enteritidis*, and *E. coli*). These three pathogens are infectious pathogens that can cause health problems and even death in poultry and cause foodborne diseases in humans after ingestion. Based on the research results, isolate F4 showed the highest inhibition zone against *S. aureus* and *E*. *coli* with inhibition zones of 15.15 mm and 22.25 mm, respectively, whereas isolate F11 showed the highest inhibition zone against *S. enteritidis* (14.50 mm). These results are higher than those reported by Mohammad *et al*. [66], who reported that the *Lactobacillus musae* SGMT17 isolate had an inhibition zone of 5 mm against *E. coli* and 5 mm against *S. aureus*. Harnentis *et al*. [1] reported an inhibition zone of 11.54 mm against *E. coli*, 10.27 mm against *S. aureus*, and 16.31 mm against *S. Enteritidis* of the N16 isolate isolated from indigenous fermented foods. Tsega *et al*. [75] also reported an inhibition zone of 17.66 mm against *E. coli*, 16 mm against *S. aureus*, and 14.50 mm against *S. Enteritidis* for the IS6 isolate isolated from the digestive tract of chicken. Winastri *et al*. [25] stated that the inhibitory power of probiotic bacteria against pathogens can be divided into weak (≤ 5 mm), moderate (6-10 mm), strong (11-20 mm), and very strong (≥ 21 mm). The tested LAB isolate in this study has the potential to be used as a probiotic because of its moderate–very strong antibacterial activity.

One of the most important criteria for probiotics is their ability to inhibit the activity of pathogenic bacteria that can infect the digestive tract of poultry. Antimicrobial ability against pathogens is a parameter that must be tested when selecting probiotic strains [76]. The antimicrobial activity of probiotic micro-organisms against pathogens plays a role in maintaining the balance of intestinal microflora and protecting the intestines from pathogens. Antimicrobial activity not only shows the ability of bacteria to inhibit pathogens, but also illustrates the ability of bacteria to help prevent disease in the host. Bacterial colonies can protect the digestive tract by attaching to the epithelial tissue on the enterocyte walls, reducing the possibility of pathogenic bacterial colonies [77].

Cell surface proteins are produced by most LABs to help them bind to intestinal epithelial cells and activate immunoregulation to protect against pathogens [78]. By adhering to the host intestine, probiotics can inhibit pathogen colonization by preventing pathogens from interacting with specific receptors on host cells or by inhibiting their anchorage through steric interactions. In addition, probiotics can produce antibacterial compounds, such as bacteriocins, which can help in resistance to certain pathogens [79]. LAB produces various organic acids such as acetic acid, lactic acid, citric acid, succinic acid, and propionic acid as end products of carbohydrate fermentation, which can inhibit the growth of pathogenic micro-organisms [80]. Organic acids such as lactic acid produced by LAB form an unfavorable microenvironment for pathogens [81]. The main objects of these organic acids are bacterial cell walls, cytoplasmic membranes, and specific bacterial metabolism that cause the destruction and death of pathogenic micro-organisms [82]. Wang *et al*. [83] reported that a lactic acid concentration of 0.5% (v/v) can prevent the growth of *Salmonella*, *E. coli*, and *Listeria monocytogenes*, which are pathogenic. The levels and types of organic acids produced by LAB are influenced by the microorganism species, culture media composition, and growth conditions [84]. Probiotic metabolites, such as EPS and SCFAs, are also involved in immune regulation, intestinal mucosal barrier protection [85], and pathogenic bacterial antagonism [86].

### Molecular identification of selected LAB isolates

Clusters A and B were derived from the phylogenetic tree shown in Figure-3. Thirteen *Lacticaseibacillus paracasei* bacteria formed cluster A. *Lacticaseibacillus casei* strain HBUAS71245, *Lactobacillus casei* strain T22, and isolate 4 are the only three bacteria found in Cluster B. Based on the phylogenetic tree and genetic distance analysis, isolate 4 has a genetic similarity of 99.76% to *Lactobacillus casei* strain *T22* (JQ412731.1) at the closest level.

The *Lactobacillus casei* group (LCG) consists of the closely related species *L. casei*, *L. paracasei*, and *L. rhamnosus* [87]. LCG is one of the most studied species due to its commercial, industrial, and applied health potential. *L. casei* is one of the most widely used LAB as a probiotic [88]. *L. casei* strains have been shown to modify the microbiota in the gut and influence the host’s immune responses [89]. The use of *L. casei* as a probiotic has been widely reported in various studies on poultry. Lokapirnasari *et al*. [90] reported that the combination of 0.5% *Bifidobacterium* spp. + 25% *L. casei* reduced FI and FCR, increased egg weight, feed efficiency, and hen-day production in laying hens compared with the control. Tabashsum *et al*. [91] stated that giving *L. casei* as a probiotic at a dose of 10^10^ CFU/mL showed resistance and could alleviate the colonization of *C. jejuni* and *S. enterica* in the cecum, jejunum, and ileum of broilers. Ju *et al*. [92] reported that recombinant *L. casei* at a dose of 1–2 × 10^9^ CFU/mL significantly increased the colonization of *Lactobacillus* and *Bifidobacterium*, reduced the relative abundance of *E. coli* in the cecum of chickens, and reduced symptoms of Newcastle disease (ND). Deng *et al*. [93] also reported the results of their research and stated that the use of *L. casei* can be a good strategy to manage intestinal inflammatory responses in chicks infected with *S. pullorum*, where administration of *L. casei* can significantly relieve diarrhea, raise daily weight gain, help in maintaining the integrity of the intestinal mucosal epithelium, successfully inhibit intestinal colonization by *S. pullorum*, and modulate the microbiota balance.

## Conclusion

*In vitro* probiotic testing to determine the best probiotic candidate showed that isolate F4 was resistant to 42°C, 94.44% to pH 2.5 for 3 h, and 83.39% to pH 2.5 for 6 h. Resistance to bile salt concentrations of 0.3% and 0.5% resulted in survival rates of 88.07% and 81.24%, respectively. Isolate F4 showed a good percentage of hydrophobicity (91.24%). This isolate also produced an antibacterial zone with an inhibitory zone diameter of 15.15 mm against *S. aureus*, 12.50 mm against *S. Enteritidis*, and 22.25 mm against *E. coli*. 16S rRNA analysis identified isolate F4 as *Lactobacillus casei* strain.

## Authors’ Contributions

MM, YM, YZ, and NN: Designed and developed the study concept. AS: Conducted the experiment, analyzed data, and wrote the original manuscript. KWL: Analyzed the data, participated in the preparation, discussion, and wrote and reviewed the manuscript. All authors have read, reviewed, and approved the submitted version of the manuscript.
